# Individualized Program with Iodine Supplementation

**DOI:** 10.1016/j.advnut.2024.100167

**Published:** 2024-01-05

**Authors:** Fengqin Wei, Chunli Liang, Xiaoti Lin

**Affiliations:** 1 From the Department of Thyroid Breast Surgery, Shanghai East Hospital, School of Medicine, Tongji University School of Medicine, Shanghai, China; 2 Department of Orthopedics, Fujian Provincial 2nd People’s Hospital, Affiliated Hospital of Fujian University of Traditional Chinese Medicine, Fuzhou, China

Dear Editor

We read with great interest the perspectives from the study by Kissock et al. [1], which advised that it is applicable to switching the global salt supply to iodized and potassium-enriched salt. We also agree with this viewpoint regarding replacement of universal salt iodization (USI) program. A double-edged sword of USI program should be taken into account.

Even data showed that USI program reduced risk of iodine deficiency disorders, for example, goiter, infant anomalies, and cognitive impairments, but excessive iodized salt, as a consequence of the mandatory USI program, significantly increases the prevalence of thyroid cancer, hyperthyroidism, and nodular goiter, which might be caused from distinctive regional social realities [2]. Moreover, recent results from the real-world study of Chinese Cancer Registry Annual Report demonstrated a set of key data, which provide a novel insight of the positive correlation between USI and thyroid diseases, for example, thyroid cancer [2]. This report indicated that the age-standardized incidence of thyroid cancer started from 3.21 in 100,000 in 2005, increased at an average annual percent change (AAPC) of 12.4%, and reached to 9.61 in 100,000 in 2015 [2]. Moreover, the age-standardized mortality of thyroid cancer was 0.30 in 100,000 in 2005, increased at an AAPC of 2.9%, and reached to 0.35 in 100,000 in 2015 [2]. Thus, the increase of incidence and mortality of thyroid cancer closely aligned with the mandatory USI program.

It has been well known that both high and low iodine intakes increased risk of thyroid disease. Mechanistic studies revealed that long-term high or low iodine intake leads to excessive secretion of serum thyroid stimulating hormone (TSH), which could trigger proliferation of thyroid follicular epithelial cells, give rise to nodular goiter, and eventually produce thyroid cancer [3]. In turn, serum TSH concentration can act as a direct and key indicator for abnormal intake of iodine and the resultant side effects. In general, the reference abnormal value accounts for 5% of the healthy population. Recently, a nationwide, cross-sectional survey of Chinese general population from 2014 to 2017 revealed that ∼13.2% of euthyroid individuals had increased concentrations of serum TSH [4]. These results verified that increased serum TSH concentration has been prevalent across China for several years with implement of mandatory USI program. Moreover, WHO recommends using urinary iodine concentration (UIC) for determining iodine status in populations. However, only using UIC to estimate the proportion of excessive iodine intake can be biased owing to high individual variability [5]. Recent investigations found that the combined use of urinary sodium concentration and UIC would alleviate the bias in evaluation of excessive iodine intake in population [5]. Therefore, determining iodine status in current populations is insufficient; it is urgent to provide precise screening of individualized iodine status for different person.

Socioeconomic factors contribute to the unsatisfactory outcome of the UIC project as well. Economic reform and marketization changed the staple supply and people’s accessibility to diversified food. It presents that more and more iodine-rich foods are consumed in daily diet, for example, kelp, seaweed, and egg [6]. Another specialty in the USI program is the strong state intervention into the salt supply where people barely have other choices. Although the adjusted program of USI has been launched in some regions, multiple coastal provinces where residents have no need of additional iodine intake are still mandated to implement the USI program. Conversely, with the mandatory USI program, more than half of pregnant women suffered from median UIC lower than WHO criterion under the USI program [7]. Therefore, we need to provide precise iodine supplement project for variable populations, although the USI cannot do so. Taken together, it is the urgent time to encourage individualized program with precise assessing and supplementing iodine.

## Author contributions

The authors’ responsibilities were as follows – FW: writing original draft; CL: review and editing; and XL: writing original draft, review, and editing.

### Conflict of interest

The authors report no conflicts of interest.

### Funding

The authors report no funding received for this study.
